# Trypsin Binding with Copper Ions Scavenges Superoxide: Molecular Dynamics-Based Mechanism Investigation

**DOI:** 10.3390/ijerph15010139

**Published:** 2018-01-15

**Authors:** Xin Li, Yongliang Zhong, Chunyan Zhao

**Affiliations:** 1 College of Food and Bioengineering, Henan University of Science and Technology, Luoyang 471023, China; tangzch@haust.edu.cn; 2 Ministry of Education Key Laboratory of Cell Activities and Stress Adaptations, Lanzhou University, Lanzhou 730000, China; 3 School of Pharmacy, Lanzhou University, Lanzhou 730000, China; zhaochy07@lzu.edu.cn

**Keywords:** trypsin, copper ions, superoxide, molecular dynamics, PCBs

## Abstract

Trypsin is a serine protease, which has been proved to be a novel superoxide scavenger. The burst of superoxide induced by polychlorinated biphenyls can be impeded by trypsin in both wild type and sod knockout mutants of *Escherichia coli*. The experimental results demonstrated that the activities of superoxide scavenging of trypsin were significantly accelerated by Cu ions. Also, with the addition of Cu ions, a new β-sheet (β7) transited from a random coil in the Cu(II)-trypsin (TP) system, which was favorable for the formation of more contacts with other sheets of trypsin. Residue–residue network analysis and the porcupine plots proved that the Cu ion in trypsin strengthened some native interactions among residues, which ultimately resulted in much greater stability of the Cu(II)-TP system. Moreover, compact and stable trypsin structures with Cu ions might be responsible for significantly provoking the activity of superoxide scavenging.

## 1. Introduction

Reactive oxygen species (ROS) are a class of ubiquitous molecules including superoxide anion (superoxide), hydrogen peroxide and hydroxyl radicals [1,2]. Reactive oxygen species regulate critical steps in signal transduction cascades and many important cellular events [2,3]. On the other hand, high levels of ROS are toxic to cells, due to their damage to cellular components [4]. In response to oxidative stress, there are alterations in the capacity of the antioxidant defense system in an attempt to minimize oxidative damage to cellular components, such as lipids, proteins and DNA [5,6]. Both eukaryotic and prokaryotic cells may protect themselves from ROS by producing antioxidant enzymes, including catalase, which degrades hydrogen peroxide, and superoxide dismutase (SOD), which dismutes superoxide [7].

Trypsin is a serine protease found in the digestive system of many vertebrates, where it hydrolyses proteins. Its excessive activity has been strongly implicated in acute pancreatitis, inflammation and tumors [8,9,10]. Trypsin has recently been proved to be a novel superoxide scavenger in intracorporal and extracorporal systems [11,12]. As an endogenous human enzyme, the superoxide scavenging activities of trypsin make it possible to act as a novel agent to eliminate the damage to cells induced by polychlorinated biphenyls (PCBs).

Furthermore, for trypsin, previous studies have proved that the ability of scavenging free radicals can be affected by adding metal ions such as calcium ions, magnesium ions and sodium ions [13,14,15]. However, the mechanism for the acceleration of superoxide scavenging activity with the addition of metal ions is unclear. It could be related to the conformation features of trypsin, considering that the base of its biological functions is the structure. As already proven, metalion complexation with proteins is a strong trigger for a structural switch of proteins [16,17,18]. Metal ion binding serves to increase or decrease the structural stability of a protein in a conformation, which is critical for a protein to perform its biological function [19]. According to several reports, the addition of metal ions could change the three-dimensional structure of trypsin; consequently, the biological function and activity of the protein are correspondingly affected [20,21,22]. Based on this, we can assume that the effect of the addition of metal ions on the ability of scavenging free radicals might be highly related to the conformational change of trypsin.

Among the metal ions, copper ions are an essential trace metal which is required for numerous metabolically important enzymes involved in various metabolic pathways of human physiology [23,24]. Many studies have shown that the binding of Cu ions to proteins leads to changes in the conformation of the copper-binding proteins [14] with a loss of irregular structure and an increase in an extended β-sheet-like conformation [25].

Thus, in the present work, trypsin with the addition of Cu ions was tested for its superoxide scavenging activity in a xanthine–xanthine oxidase superoxide producing system. Also, molecular simulation approaches were carried out to characterize the structural details of Cu ions on trypsin structures on a molecular level.

## 2. Materials and Methods 

### 2.1. Bacteria 

The *Escherichia coli* wild type strain AB1157 and *sod* knockout mutant [PN134, (sodA::Mud PR13) 25, (sodB-Kan)1-Δ2] used in our work were kindly supplied by James A. Imlay (Department of Microbiology, University of Illinois at Urbana-Champaign, IL, USA). A single colony was inoculated into a LB liquid medium (containing Tryptone 10 g/L, Yeast Extract 5 g/L, NaCl 10 g/L) to obtain an overnight pre-culture. Strains were diluted from the overnight culture and incubated at 37 °C for four to six generations. Cultures of the strains were diluted to approximately 10^9^ cells per mL to be used in this study.

### 2.2. Polychlorinated BiphenylsTreatment 

5 μM PCBs including PCB 153, PCB 180, PCB 11 and 4-OH-PCB 11 were added in the culture of *E. coli* and shaken continuously at 37 °C for 30 min [26].

### 2.3. Trypsin Treatment

Trypsin (bovine, 500 units/mg, crystalline) was purchased from Amersco (Solon, OH, USA). Trypsin (2.14 × 10^−7^ mol/L) was added in the culture strains. The mixture was then incubated at 37 °C for 30 min.

### 2.4. Quantitative Assay of Superoxide Anion

Superoxide concentration in the bacteria culture was measured by electron spin resonance (ESR) as described by us [19]. Levels of superoxide in bacterial cells were determined by ESR measurements with 1,2-dihydroxybenzene-3,5-disulfonic acid (Tiron, from Sigma-Aldrich, Hong Kong, China) [27,28]. Tiron reacts with superoxide to form the Tiron semiquinone, which is detectable by ESR as a four-line first derivative spectrum. The stability and specificity of the signal of Tiron with superoxide has been confirmed by SOD addition. In our experiments, Tiron was added to all the preparations to a final concentration of 10 mM. The signal for superoxide was determined by ESR spectrometer (Bruker ER 200 D, Karlsruhe, Germany) as described by us previously in [19].

### 2.5. External Factors Treatments

Samples were incubated in 0.5 mM Cu^2+^ at 28 °C for 0.5 h in accordance with the method described by Takahama et al. [29]. Soybean trypsin inhibitor (STI, Protein Data Bank Identification Code (PDB ID: 1AVU), Sigma-Aldrich, Hong Kong, China) was added to the fertilization medium resulting in a final concentration of 5 µM and incubated with bacterial cells at 28 °C for 0.5 h.

### 2.6. Molecular Dynamics Simulation

The structure of trypsin was obtained from RCSB Protein Data Bank (RCSB PDB) with PDB ID 4F5S [30]. The three complexes of trypsin binding with no ligand (APO-TP), with Cu ions (Cu(II)-TP) and with inhibitor soybean trypsin (inhibitor-TP) were built using Discovery Studio 2.0 software (DS 2.0, BIOVIA Company, San Diego, CA, USA). All molecular dynamics simulations were performed using the GROMACS 5.0 (http://www.gromacs.org/), utilizing the all-atom 53A6 force field [31,32]. The topology was generated using the pdb2gmx tool with standard pH 7 amino acid protonation state. The grid preparation was done using the extended simple point charge (SPC/E) solvent model. The protein with added ions was centered in a cubic box of dimensions specifying a solute-box distance of 1.0 nm with SPC/E water molecules. Then, the minimized complexes were submitted to 100 ps of molecular dynamic (MD) at 300 K, followed by a steep descent, and conjugate gradients until an energy of 1000.00 kJ·mol^−1^·A^−1^, considering constant volume/constant temperature (NVT) and constant pressure/constant temperature (NPT) state with position restrained to the entire system using the V-rescale thermostat and the Parrinello–Rahman scheme for pressure coupling. Then, dynamics simulations of the full system (protein and water) without any positional restraints were then applied with convergence criteria of 1000.00 kJ·mol^−1^·A^−1^. Finally, the entire scheme was subjected to MD production at a temperature of 300 K for 50 ns.

Residue–residue network analysis was performed by Cytoscape 3.4.0 and solvent-accessible surface area (SASA) analysis by CASTp (http://sts.bioe.uic.edu/castp/). Principal component analysis (PCA) and porcupine plots were applied by VMD1.9.2 (University of Illinois, Urbana-Champaign, IL, USA) and dynamical cross-correlation maps by Prody 1.5.1 (http://prody.readthedocs.io/en/latest/about/index.html).

### 2.7. Statistics 

SPSS for Windows 11.5 was used for statistical analysis. Results are reported as mean ± standard error of the mean (SEM) The significance of differences between superoxide anion affected by Cu^2+^ or STI was determined using a paired-sample *T*-test. Values are denoted as significant (*p* < 0.05) or highly significant (*p* < 0.01).

## 3. Results

### 3.1. Effects of Metals on Trypsin Activity

**Figure 1:**
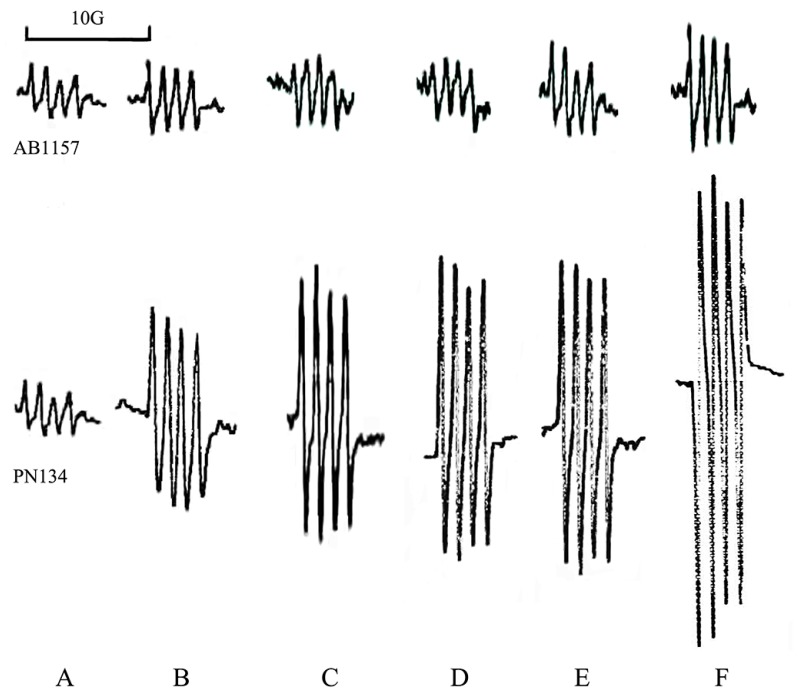
Effect of polychlorinated biphenyls (PCBs) on the electron spin resonance (ESR) spectrum of superoxide in the culture of *Escherichia coli*. AB1157 is a wild type strain; PN134 is a *sod* mutant. Bar, 10G. (**A**) Medium control; (**B**) Culture of strains; (**C**) Culture with PCB153; (**D**) Culture with PCB180; (**E**) Culture with PCB11; (**F**) Culture with 4-OH-PCB11.

**Figure 2:**
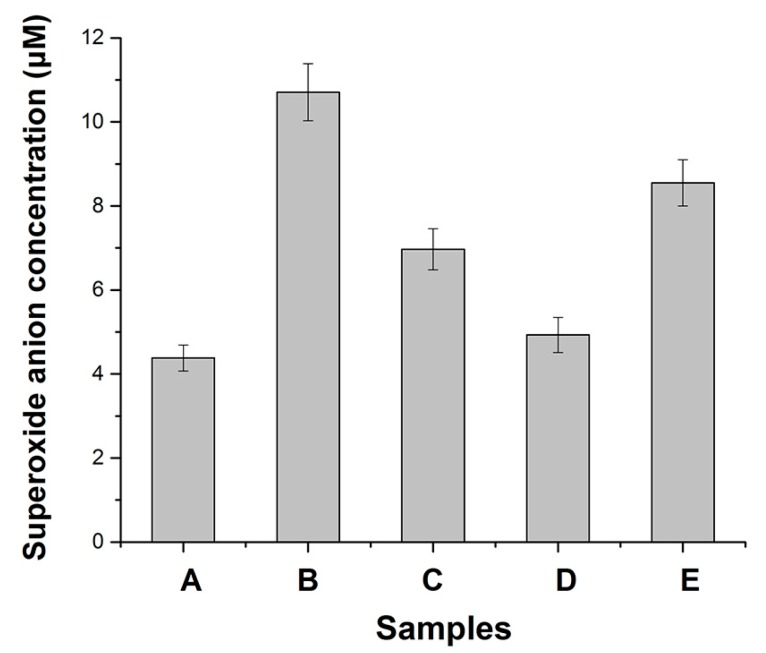
Inhibition of Cu^2+^ or soybean trypsin inhibitor (STI) on trypsin activities of superoxide scavenging in *sod* mutant strain PN134. (**A**) Culture of PN134; (**B**) Culture of PN134 with 4-OH-PCB11; (**C**) Culture of PN134 with 4-OH-PCB11 plus trypsin; (**D**) Culture of PN134 with 4-OH-PCB11 plus trypsin and Cu^2+^; (**E**) Culture of PN134 with 4-OH-PCB11 plus trypsin and inhibitor STI.

Reproducible results obtained from three or more independent ESR assays suggested that superoxide was observed in the culture of either wild type strain AB1157 or *sod* mutant PN134 (Figure 1B). The superoxide signal of PN134 was much higher than that of AB1157. The LB medium control also produced a small ESR signal in the presence of Tiron (Figure 1A). In our previous work, the amplitude of the Tiron signal was reduced by more than 95% with SOD addition (200 units per mL), confirming that the ESR spectrum had been derived from superoxide [28]. In either wild type or sod mutant strains, the superoxide signal was increased with the treatment of PCB153, PCB180, PCB11 or 4-OH-PCB 11 (Figure 1C–F). Among the PCBs, 4-OH-PCB11 significantly increased the level of endogenous superoxide of *E. coli* (Figure 1F). Consistent with the result of the ESR, and highly significantly, endogenous superoxide of PN134 cells stimulated by 4-OH-PCB 11 jumped to more than 2 folds of control (Figure 1B, *p* < 0.01). Results showed that trypsin scavenged the endogenous superoxide in *E. coli* (Figure 1C, *p* < 0.01). The superoxide scavenging activities of trypsin were significantly accelerated by Cu^2+^ (Figure 1D, *p* < 0.05), while also being significantly impeded by STI (Figure 1E, *p* < 0.05). Previously, we observed trypsin scavenging superoxide in vitro. When trypsin was incubated with culture of *E. coli*, the excess superoxide caused by 4-OH-PCB11 was definitely impeded. In the current work, the superoxide scavenging activity of trypsin was also significantly accelerated by Cu^2+^ and partly eliminated by the inhibitor STI in *sod* mutant strain PN134 (Figure 2). 

According to several reports [25], the addition of metal ions could have an effect on the three-dimensional structure of trypsin. As a consequence, the biological function and activity of the protein would be correspondingly affected. Therefore, we assume that the addition of Cu ions will change the structure of trypsin, which might be the reason for the acceleration of trypsin. Thus, as described below, molecular dynamics modeling was applied to make clear the structural effects of trypsin induced by the addition of Cu ions on a molecular level.

### 3.2. Model Construction

**Figure 3:**
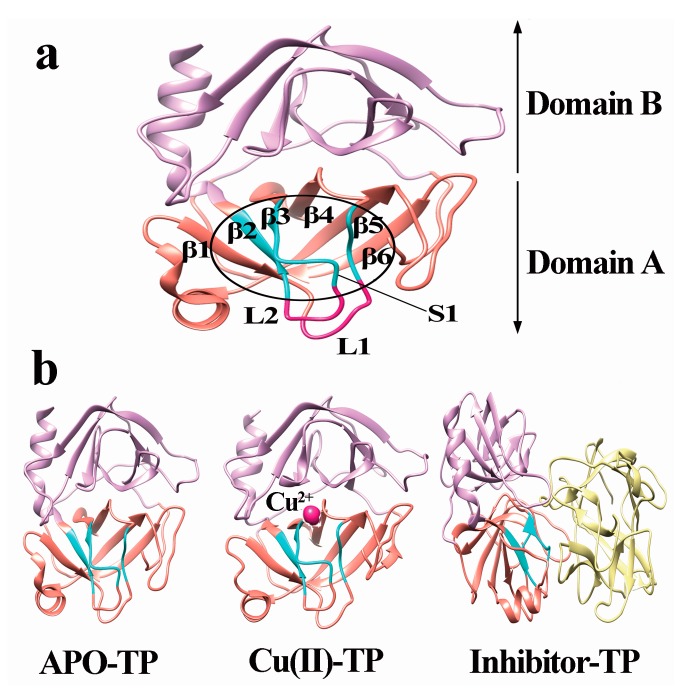
Three dimensional of trypsin protein. (**a**) Structure of S1 pocket; (**b**) Structure of APO-TP, Cu(II)-TP and Inhibitor-TP. Domain A of trypsin is shown as purple ribbon and domain B as golden ribbon. Active site residues of trypsin are shown with β-sheets in blue (β2, β3, β4, β5 and β6) and loops (L1 and L2) in magenta. Cu ions are presented as a deep pink ball and STI is presented as golden ribbon.

As presented in Figure 3, the structure of trypsin includes domain A and domain B, each of which consists of six reverse parallel β-sheets. The six β-sheets are named as: β1 (Met180-Ala183), β2 (Gly226-Thr229), β3 (Lys204-Trp215), β4 (Pro198-Cys201), β5 (Gln135-Gly140), and β6 (Lys156-Pro161) in domain A (Figure 3a). Residues Asp189-Ser195, Ser214-Cys220 and Pro225-Tyr228 form the primary substrate-binding pocket called the S1 binding pocket. Residues Leu185-Gly188 and Ala221-Lys224 form two loops near the S1 pocket, called L1 and L2, respectively. Residues Ser217-Gly219 are generally considered as the lid of the S1 pocket, which controls the entrance of substrate [33].

In the present work, we constructed three different systems, including trypsin binding with no ligand (APO-TP), with Cu ions (Cu(II)-TP) and with inhibitor soybean trypsin (inhibitor-TP) as shown in Figure 3b. The structures of these three systems were then subject to molecular dynamics modeling. 

The overall structural stability throughout the molecular simulation was controlled by root-mean-square deviations (RMSD) over time (Supplementary Figure S1). As shown, all three systems reached a stable state in simulations and the average RMSD fluctuation values remained at 2.1 Å, 3.1 Å and 3.7 Å. Root-mean-square fluctuation (RMSF) of the Cα atoms of all the residues were then plotted to determine the behavior of residues (Supplementary Figure S2). As shown, the three systems (APO-TP, Cu(II)-TP and Inhibitor-TP) shared similar RMSF distributions and similar trends in their dynamic features. However, it should be noted that the Cu(II)-TP system presented the lowest average RMSF value of 1.12 Å compared with the APO-TP system of 1.65 Å and Inhibitor-TP system of 2.47 Å. This illustrates that Cu ions binding with trypsin results in a stable trypsin structure.

### 3.3. Residue–Residue Network Analysis

**Figure 4:**
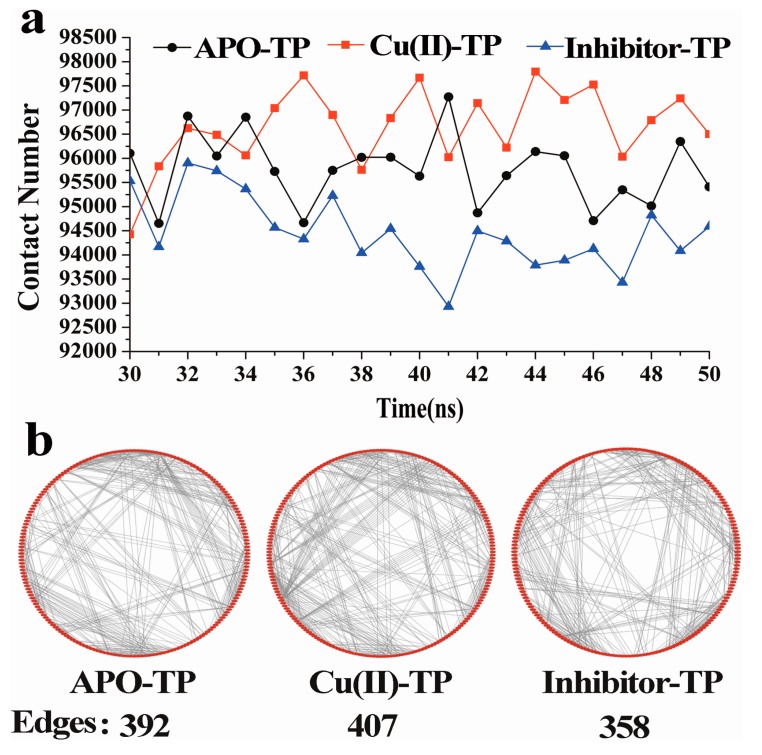
The contacts and residue-residue network of three systems. (**a**) The number of the contacts in trypsin as a function of time during the simulations for APO-TP model (black), Cu(II)-TP model and inhibitor-TP model (blue); (**b**) Residue-residue network of three systems. Residues are represented as red dots evenly distributed around the circle, while interactions between residues including hydrogen-bond interactions, van der Waals force, ionization and π-π stack are defined as edges represented by grey lines.

The contacts between residues within a distance of <0.6 nm were calculated and plotted in Figure 4a for the three systems as a function of simulation time. The contact number within the protein provides a way to estimate the compactness of the structure of biomolecules, which also depends upon its hydrophobicity. As shown, the average numbers of contacts for the Cu(II)-TP, APO-TP and inhibitor-TP systems were 96,660, 95,770 and 94,459, respectively. Many more contacts formed in the Cu(II)-TP system during the simulation process, which suggests that the structure of Cu(II)-TP is more compact.

On the other hand, the intra-residue interactions within trypsin were represented by a topology based network, which could be considered as a novel strategy for identifying the overall stability of protein (Figure 4b). Residues are represented as red dots evenly distributed around the circle in a clockwise direction, while interactions between residues, including hydrogen-bond interaction, van der Waals force, ionization and π-π stack are defined as edges represented by grey lines [34]. As shown, total numbers of 407, 398 and 358 edges were presented within residues of the Cu(II)-TP, APO-TP and inhibitor-TP systems, respectively. The highest number of edges is in the Cu(II)-TP system, which generally indicates the most compact protein tertiary structure.

All evidence proved that the complexing of Cu ions with trypsin strengthened some native interactions among residues, which ultimately resulted in much more stability of the Cu(II)-TP system. 

### 3.4. Hydrogen Bonds Analysis 

**Figure 5:**
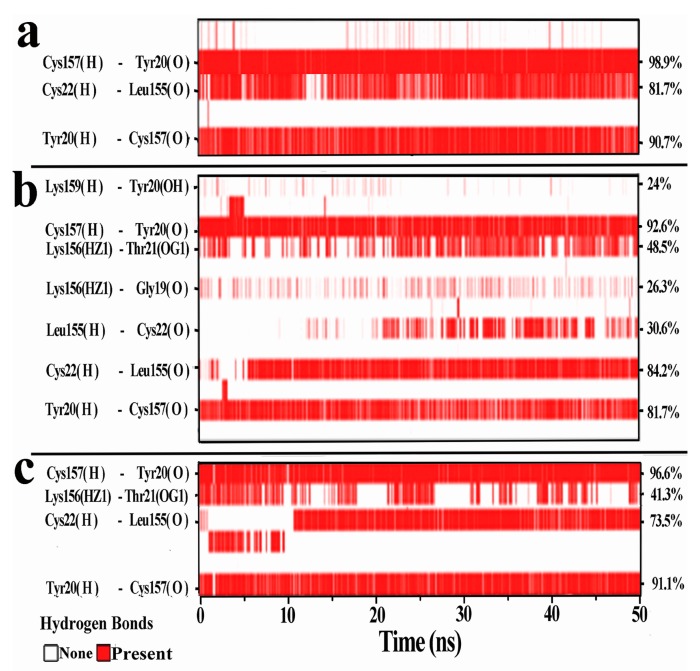
Hydrogen bond existence map between residues of β6 and β7. The red band shows the existence of hydrogen bonds while the white band shows the absence of hydrogen bonds. Donor/acceptor pairs are shown for (**a**) APO-TP; (**b**) Cu(II)-TP; and (**c**) Inhibitor-TP systems. Only hydrogen-bond pairs that have an incidence greater than 20% in the WT system are shown.

As shown in Figure 5, there was a new β-sheet named β7 constructed from the random coil of residues Gly19-Cys22 in the Cu(II)-TP system. It has been reported that a conformational transition of protein from random and/or helical coil to β-sheet is generally correlated with metallic ion binding [35]. The reconstruction of β-sheet in Cu(II)-TP systems indicated more stable and more regular conformations. In addition, the transition to β-sheet was beneficial for forming more contacts with other sheets, including hydrogen-bond interaction, van der Waals force, ionization and π-π stacking. Among these contacts, hydrogen bonds are generally believed to be the driving force for maintaining the stability of β-sheets in trypsin [36]. Thus, analysis of hydrogen bonds (H-bonds) between all β-sheets was approached by using existence maps (Supplementary Figures S3–S6) and hydrogen bond occupation tables (Supplementary Tables S1–S4) to confirm the governing forces of the different systems.

On the whole, the Cu(II)-TP system presented the largest number and the highest occupation of H-bond pairs. It also can be observed that the most distinctive features of H-bonds were found to be between β1-β2, β2-β3, β5-β6 and β6-β7 in all three complexes of APO-TP, Cu(II)-TP and inhibitor-TP, especially between β6 and β7. For the H-bonds between β6 and β7, a group of 7 specific hydrogen bonds formed in the APO-TP systems, while the values of H-bonds in the Cu(II)-TP and Inhibitor-TP complexes were 3 and 4. The addition of intermolecular hydrogen bonds in the Cu(II)-TP complex included four H-bonds of Lys159@H-Tyr20@OH, Lys156@HZ1-19Gly@O, Lys156@HZ1-Thr21@OG1 and Leu155@H-Cys22@O, which accounted for the assembly of the newly formed β-sheet structure within these molecules. Also, the most significant change in H-bonds was in residues Tyr20, Leu155 and Lys156. Considering that Tyr20 was one of the residues of the newly formed β7 coming from a loop of Gly19-Cys22, hydrogen interactions between Tyr20 and residues in β6 might play a key role in transformation from loop to β-sheet. In addition, the loop region of Gly19-Cys22 was turning from the end of the domain A to a position forming more contacts with β6. This also helps to make the whole structure of protein more compact. 

Taken together, construction of the new β-sheet facilitated more contacts between all β-sheets and made the conformation more regular as a consequence of the Cu ions. 

### 3.5. Radius of Gyration and SASA Analysis

**Figure 6:**
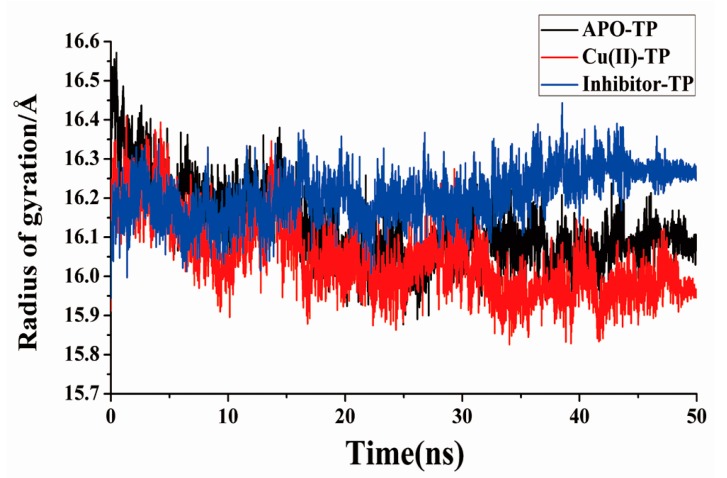
Radius of gyration (Rg) of the APO-TP, Cu(II)-TP and Inhibitor-TP models.

The radius of gyration (Rg) was calculated to further clarify the degree of conservation of the structure. For further clarification of the degree of conservation of the trypsin structure with Cu ions, the temporal changes in the SASA of the substrate binding pocket were calculated for all three independent trajectories. Rg is defined as the mass-weight root mean square distance of the collection of atoms from their common center of mass, which provides a way to estimate the compactness of structures of biomolecules [37]. As shown in Figure 6, as the simulation proceeded, the Rg value of all three complexes decreased and stabilized at a minimum value, representing the equilibrium state. As seen, the value of Rg for the Cu(II)-TP system shows a minimum of 15.96 Å, comparable with the APO-TP system. The Rg value of the Inhibitor-TP system showed the higher value (Rg = 16.40 Å) in the final period compared with the APO-TP system (Rg = 16.13 Å). This indicates that the conformation of TP protein when combined with Cu ions tends to be more stable and compact.

On the other hand, as shown in Table 1, the SASA of hydrophobic residues in Cu(II)-TP, APO-TP and inhibitor-TP systems presented a significant decreasing trend with values of 85.36 Å^2^, 53.68 Å^2^ and 32.11 Å^2^, respectively. As known, the decreasing SASA was generally related to the changing solvent exposure of residues in the different conformations, and might have occurred due to the stability lost in the protein structure.

Namely, the main variation in hydrophobic binding sites could make the hydrophobic residues more readily accessible to the solvent molecule. Thus, the highest SASA value signified that more stabilizing interactions (intermolecular hydrogen bond, electrostatic and van der Waals interactions) could gradually occur in the Cu(II)-TP system, as compared to the other two systems. 

### 3.6. Principal Component Analysis (PCA) and Porcupine Plots

**Figure 7:**
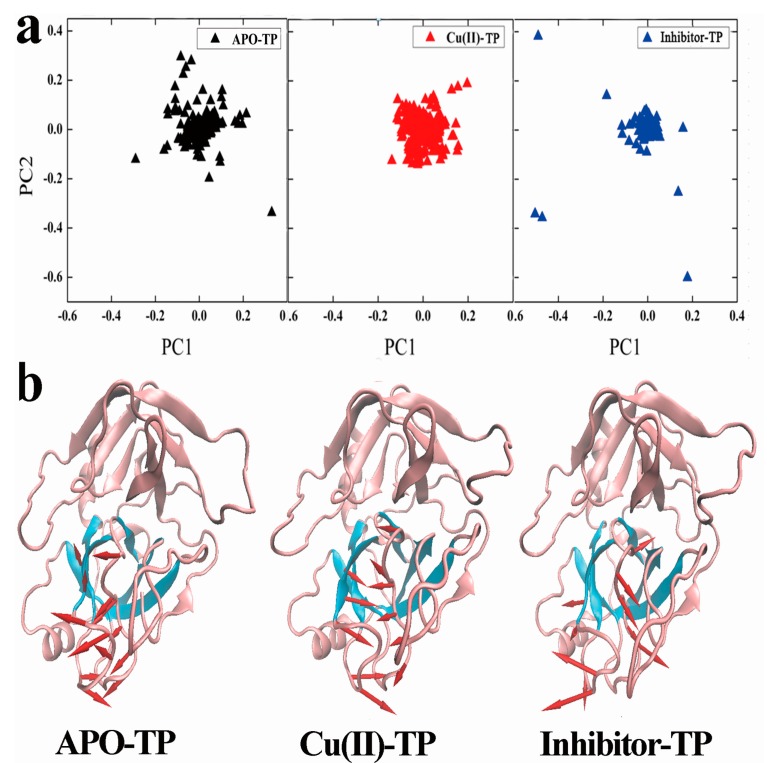
(**a**) The plots of the first two principal components (PC1, PC2) calculated from structures of the three MD trajectories. The APO-TP model is presented in black triangles, the Cu(II)-TP model in red triangles and the inhibitor-TP model in blue triangles; (**b**) The porcupine plot of the first eigenvector of the APO-TP model, Cu(II)-TP model and inhibitor-TP model. The arrows indicate the direction of eigenvector and magnitude of the corresponding value. The significant motion mode of the S1 binding pocket and the two loops outside the pocket is shown as magenta arrows.

Essential dynamics sampling was performed based on two major principal components (i.e., PC1 and PC2) to find the conformational subspaces during dynamics (Figure 7a) [38]. The two principal components (named, PC1 and PC2) represented more than 20% of the total sample variance for the three sets of structures of the three systems. Each distribution of dots represents the conformational subspace sampled by the respective molecule. It was apparent that the APO-TP system distributed in a much larger region in the conformational space, while the projection distribution of Cu(II)-TP is more concentrated in a smaller range. This indicated that protein underwent conformational changes which made the structure of trypsin became compact and tight upon binding with Cu ions. The distribution of the inhibitor-TP system was quite broad, which proved that the structure of trypsin was loose and inflatable upon binding with inhibitor.

Additionally, porcupine plots of the top eigenvectors were carried out for each system (Figure 7b). As seen, the residues of the S1 binding site in the Cu(II)-TP system were moving toward the center of the pocket, leading to the formation of a much more compact structure with the edges of the pocket facing each other. At the same time, the stretched inhibitor-TP system shared a prominent inflation pulling each other in opposite directions, which was also a reflection of the overall conformational expansion.

### 3.7. Dynamical Cross-Correlation Maps and Distance Analysis

**Figure 8:**
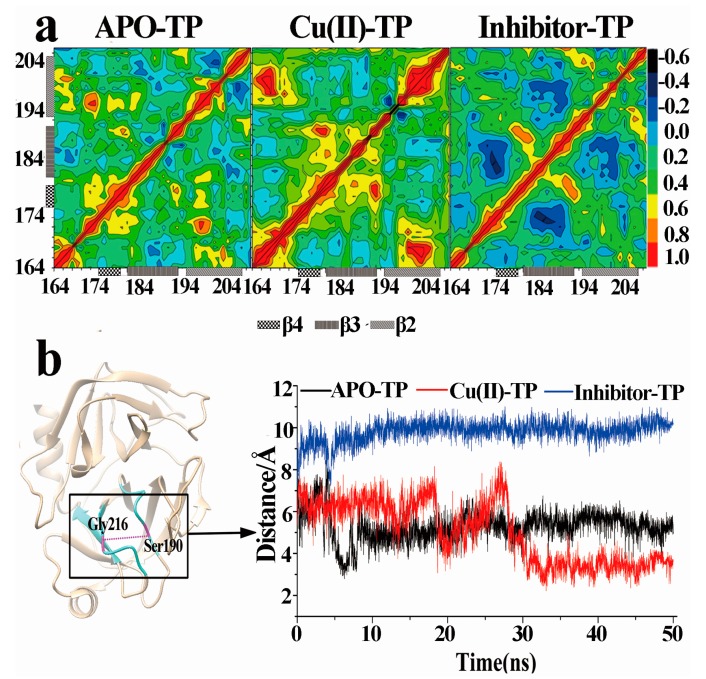
Dynamical cross-correlation maps and distance fluctuation plot. (**a**) Dynamical cross-correlation maps (DCCM) of S1 pocket residues for the APO-TP, Cu(II)-TP and inhibitor-TP models. The correlation efficient between two residues is represented by the color map shown on the right side. Blue represents the strong anticorrelated motions and red represents the strong correlated motions; (**b**) The distance fluctuation plot between Cα atoms of Ser190 and Gly216 for the three systems as a function of simulation time. The distance measures the open degree of S1 pocket in the trypsin. The S1 pocket is colored cyan and the two residues are colored magenta.

Further, we checked the dynamical cross-correlation of all residues for the three systems (Figure 8a). For the Cu(II)-TP complex, the results showed that the cross-correlation map as a whole, had a strong correlated motion with many regions, especially with the front part of the lid region, Gln192-Tyr206 (residue Ser214-Tyr228), and a strong anticorrelated motion with the bottom part of the lid region, Ala171-Thr177 (residue Asp189-Ser195). These motions make the pocket close and the conformation of trypsin tighter. Similar to the Cu(II)-TP system, the dynamical cross-correlation of these regions in the APO-TP system showed comparable correlative relationships. The correlation was weak, especially between region Ser150-Thr207 and the region Asn100-Ser150. Contrary to the APO-TP and Cu(II)-TP systems, these regions mostly presented a highly negative correlation relationship in the inhibitor-TP system. The region Ala171-Thr177 and region Gln192-Tyr206 moved outward in opposite directions, which made the distribution much larger and more disperse. 

On the other hand, the distance between Cα atoms of Ser190 and Gly216 are an indicator of degree of openness of the protein conformation in the S1 pocket (Figure 8b). This distance begins from the lid region, passes through active site and finally ends at an edge of the S1 pocket. As shown, for the Cu(II)-TP system, there was a substantial fluctuation in this distance within the first 25 ns and the plot was maintained at an average distance at about 5 Å after a dramatic decrease. In the APO-TP system, after an initial precipitous decrease, there was middle fluctuation in the distance of 7 Å through the remainder of the simulation period. As a comparison, the distance of the inhibitor-TP system remained essentially constant with an average distance of 12 Å during the simulation period. 

As mentioned above, the overall motion of the S1 pocket in the Cu(II)-TP system showed an obvious centralizing tendency, which made the S1 pocket much tighter together with the smaller distance of Ser190 and Gly216. These motions resulted in more compact and tight structure of trypsin in the Cu(II)-TP system.

## 4. Discussion

In the present work, experiments proved that the superoxide scavenging activity of trypsin could be significantly accelerated by addition of Cu ions in a xanthine-xanthine oxidase superoxide producing system. Molecule modeling approaches further provided the structural evidence proving that the increased activity could be explained by the increasing stability of trypsin induced by Cu ions. 

In detail,

(1) The activities of superoxide scavenging of trypsin were significantly accelerated by Cu ions.
(2) A new β7 sheet transited from a random coil in the Cu(II)-TP system, which was favorable for forming more contacts with other sheets, including hydrogen-bond interactions. It indicated that trypsin underwent conformational changes which made the structure of trypsin much compact and tight upon binding with Cu ions.
(3) Residue-residue network analysis demonstrated that many more contacts formed during the simulation process in the Cu(II)-TP system. In addition, the decreased Rg and increased SASA of the Cu(II)-TP system showed that more stable interactions gradually formed. It proved that Cu ions in trypsin strengthened some native interactions among residues, which finally resulted in much greater stability of the Cu(II)-TP system.
(4) PCA analysis and the porcupine plot for the Cu(II)-TP system showed that the projection distribution of Cu(II)-TP was more concentrated in a smaller range, with the movement of residues of S1 sites toward the center of the pocket. This led to the formation of a much more compact S1 pocket together with a smaller distance for Ser190 and Gly216.

## 5. Conclusions

This study confirmed that Cu ion binding with trypsin results in conformational changes, which ultimately makes the trypsin structure more stable. It also explains that the addition of Cu ions to trypsin can significantly provoke the activity of superoxide scavenging.

## Figures and Tables

**Table 1:** Average value of SASA.

SASA (Å^2^)	APO-TP	Cu(II)-TP	Inhibitor-TP
All residues	104.87	167.29	76.92
Hydrophobic Residues	53.68	85.36	32.11

## Supplementary Materials

The following are available online at www.mdpi.com/1660-4601/15/1/139/s1, Figure S1: RMSD values of different three systems APO-TP, Cu(II)-TP and inhibitor-TP, Figure S2: Cα RMSF per residue of different three systems APO-TP, Cu(II)-TP and inhibitor-TP, Figure S3: Hydrogen bond existence map between residues of β1 and β2, Figure S4: Hydrogen bond existence map between residues of β2 and β3, Figure S5: Hydrogen bond existence map between residues of β3 and β4, Figure S6: Hydrogen bond existence map between residues of β5 and β6, Table S1: H-bonds pairs formed between β1 and β2, Table S2: H-bonds pairs formed between β2 and β3, Table S3: H-bonds pairs formed between β4 and β5, Table S4: H-bonds pairs formed between β5 and β6.
