# Supplementary material for: Trypsin Binding with Copper Ions Scavenges Superoxide: Molecular Dynamics-Based Mechanism Investigation
Source: Int J Environ Res Public Health. 2018 Jan 15;15(1):139. doi: 10.3390/ijerph15010139 (PMC5800238; doi:10.3390/ijerph15010139)
Supplement: Supplementary file 1 [file ijerph-15-00139-s001.pdf]

molecules

Supplementary Material: Trypsin Binding with Copper Ions Scavenges Superoxide: Molecular Dynamics-Based Mechanism Investigation

Xin Li [1,2,*], Yongliang Zhong [1] and Chunyan Zhao [3]

[1] College of Food and Bioengineering, Henan University of Science and Technology, Luoyang 471023, China; tangzch@haust.edu.cn
[2] Ministry of Education Key Laboratory of Cell Activities and Stress Adaptations, Lanzhou University, Lanzhou 730000, China
[3] School of Pharmacy, Lanzhou University, Lanzhou 730000, China; zhaochy07@lzu.edu.cn
* Correspondence: lixin@haust.edu.cn; Tel.: +86-0379-6428-2342

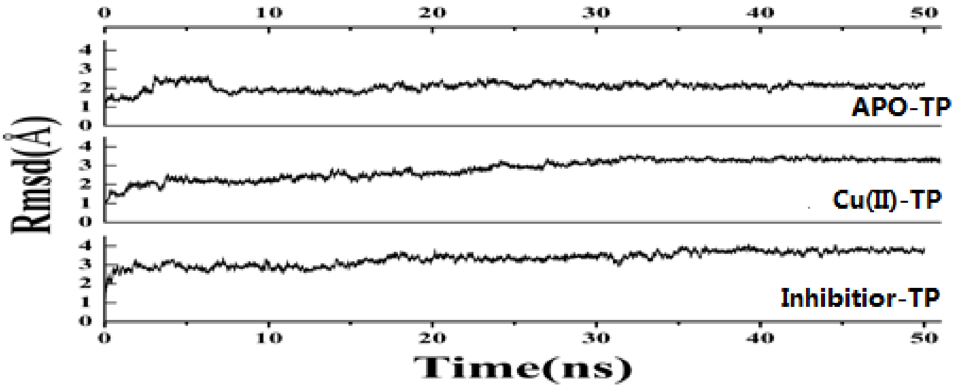

Figure S1. RMSD values of different three systems APO-TP, Cu(II)-TP and Inhibitor-TP.

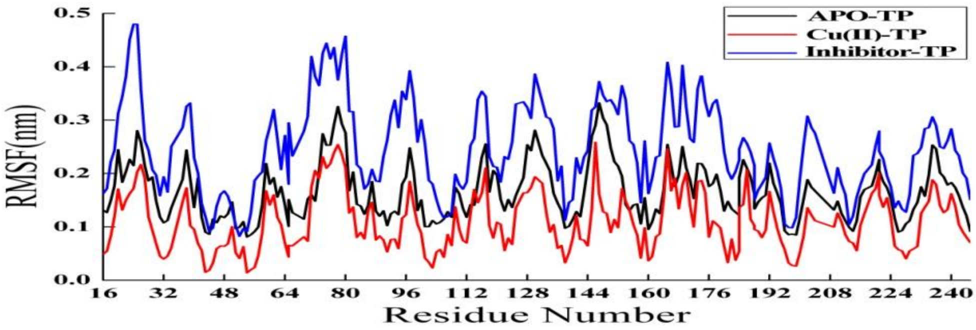

Figure S2. Cα RMSF per residue of different three systems APO-TP, Cu(II)-TP and Inhibitor-TP.

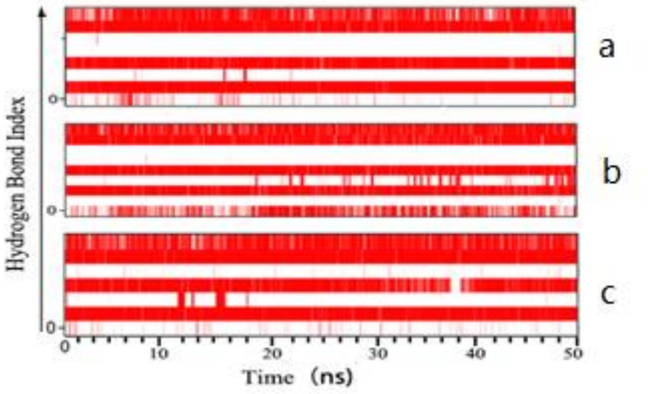

Figure S3. Hydrogen bond existence map between residues of β1 and β2. Red band shows existence of hydrogen bond while white band shows absence of hydrogen band. Donor/acceptor pairs are shown for (a) APO-TP (b) Cu(II)-TP and (c) Inhibitor-TP systems. Only hydrogen-bond pairs that have an incidence greater than 20% in the WT system are shown.

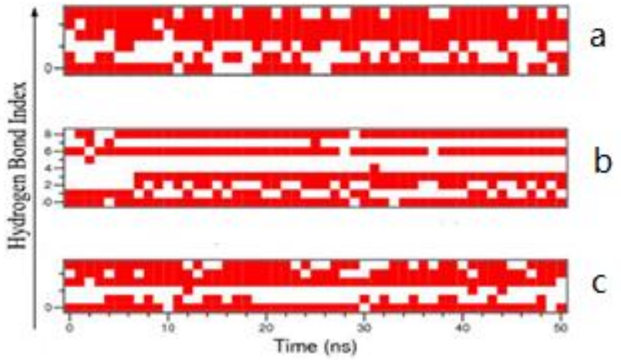

Figure S4. Hydrogen bond existence map between residues of β2 and β3. Red band shows existence of hydrogen bond while white band shows absence of hydrogen band. Donor/acceptor pairs are shown for (a) APO-TP (b) Cu(II)-TP and (c) Inhibitor-TP systems. Only hydrogen-bond pairs that have an incidence greater than 20% in the WT system are shown.

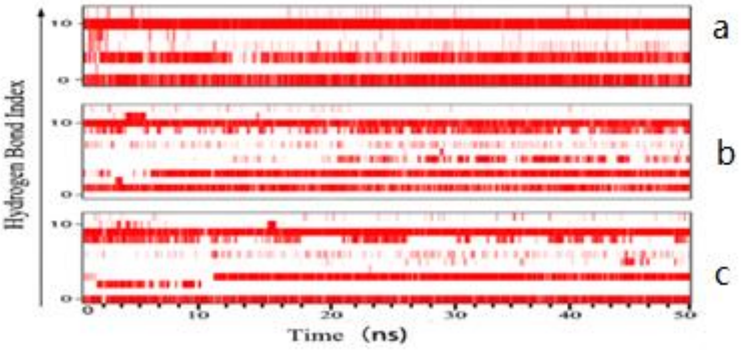

Figure S5. Hydrogen bond existence map between residues of β3 and β4. Red band shows existence of hydrogen bond while white band shows absence of hydrogen band. Donor/acceptor pairs are shown for (a) APO-TP (b) Cu(II)-TP and (c) Inhibitor-TP systems. Only hydrogen-bond pairs that have an incidence greater than 20% in the WT system are shown.

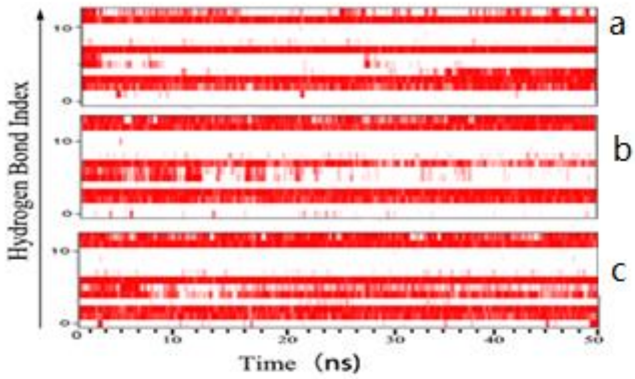

Figure S6. Hydrogen bond existence map between residues of β5 and β6. Red band shows existence of hydrogen bond while white band shows absence of hydrogen band. Donor/acceptor pairs are shown for (a) APO-TP (b) Cu(II)-TP and (c) Inhibitor-TP systems. Only hydrogen-bond pairs that have an incidence greater than 20% in the WT system are shown.

Table S1. H-bonds pairs formed between β1 and β2

	No.	H-bonds Pairs	Donor-Acceptor	Occupation (%)
APO-TP	1	1941 - 1510	230LYS(HZ1) - 180MET(O)	0.1
	2	1498 - 1945	179ASN(D21) - 230LYS(O)	0.2
	3	1921 - 1541	228TYR(HH) - 183ALA(O)	1.1
	4	1894 - 1541	226GLY(H) - 183ALA(O)	8.3
	5	1537 - 1897	183ALA(H) - 226GLY(O)	82.3
	6	1934 - 1501	230LYS(H) - 179ASN(O)	97.1
	7	1512 - 1923	181PHE(H) - 228TYR(O)	98.5
	8	1907 - 1527	228TYR(H) - 181PHE(O)	99.4
Cu(II)-TP	1	1498 - 1945	179ASN(D21) - 230LYS(O)	3.2
	2	1894 - 1564	226GLY(H) - 184TYR(O)	8.6
	3	1941 - 1510	230LYS(HZ1) - 180MET(O)	10.1
	4	1921 - 1541	228TYR(HH) - 183ALA(O)	11.1
	5	1894 - 1541	226GLY(H) - 183ALA(O)	59.4
	6	1537 - 1897	183ALA(H) - 226GLY(O)	91.1
	7	1512 - 1923	181PHE(H) - 228TYR(O)	95.8
	8	1907 - 1527	228TYR(H) - 181PHE(O)	96.3
	9	1934 - 1501	230LYS(H) - 179ASN(O)	96.9
Inhibitor-TP	1	1941 - 1510	230LYS(HZ1) - 180MET(O)	1
	2	1894 - 1541	226GLY(H) - 183ALA(O)	4.2
	3	1921 - 1541	228TYR(HH) - 183ALA(O)	4.4
	4	1537 - 1897	183ALA(H) - 226GLY(O)	87.7
	5	1934 - 1501	230LYS(H) - 179ASN(O)	92.3
	6	1907 - 1527	228TYR(H) - 181PHE(O)	98.7
	7	1512 - 1923	181PHE(H) - 228TYR(O)	99.1

Table S2. H-bonds pairs formed between β2 and β3

	No.	H-bonds Pairs	Donor-Acceptor	Occupation (%)
APO-TP	1	1785 - 1905	215TRP(H)- 227VAL(O)	72.5
	2	1760 - 1932	212ILE(H) - 229THR(O)	96.1
	3	1929 - 1780	229THR(HG1)- 214SER(OG)	94.1
	4	1925 - 1780	229THR(H) - 214SER(OG)	31.4
	5	1925 - 1767	229THR(H) - 212ILE(O)	37.3
	6	1899 - 1804	227VAL(H) - 215TRP(O)	82.4
Cu(II)-TP	1	1785- 1905	215TRP(H) - 227VAL(O)	92.2
	2	1777- 1905	214SER(H) - 227VAL(O)	59
	3	1760- 1932	212ILE(H) - 229THR(O)	94.1
	4	1760- 1928	212ILE(H) - 229THR(OG1)	60
	5	1941- 1748	230LYS(HZ1)-210GLN(OE1)	60
	6	1929- 1780	229THR(HG1)-214SER(OG)	86.3
	7	1925- 1780	229THR(H) - 214SER(OG)	47.1
	8	1925- 1767	229THR(H) - 212ILE(O)	45.1
	9	1899- 1804	227VAL(H) - 215TRP(O)	94.1
Inhibitor-TP	1	1785 - 1905	193TRP(H) - 205VAL(O)	72.5
	2	1760 - 1932	190ILE(H) - 207THR(O)	80.4
	3	1929 - 1780	207THR(HG1)-192SER(OG)	76.1
	4	1925 - 1780	207THR(H) - 192SER(OG)	5.9
	5	1925 - 1767	207THR(H) - 190ILE(O)	31.4
	6	1899 - 1804	205VAL(H) - 193TRP(O)	74.1

Table S3. H-bonds pairs formed between β4 and β5

	No.	H-bonds Pairs	Donor-Acceptor	Occupation (%)
APO-TP	1	58 -1280	22CYS(H) - 155LEU(O)	78.1
	2	31 -1301	20TYR(H) - 157CYS(O)	90.7
	3	1295- 47	157CYS(H) - 20 TYR(O)	98.9
Cu(II)-TP	1	66 - 1280	23GLY(H) - 155LEU(O)	12.1
	2	31 -1294	20TYR(H) - 157CYS(N)	14.2
	3	1273 - 69	155LEU(H) - 23 GLY(O)	17.2
	4	53 - 1280	21THR(HG1)- 155LEU(O)	18.1
	5	1319 -44	159LYS(HZ1)-20TYR(OH)	19.6
	6	1312 -44	159LYS(H) -20 TYR(OH)	24
	7	1289 - 29	156LYS(HZ1) -19 GLY(O)	26.3
	8	1273 - 64	155LEU(H) - 22 CYS(O)	30.6
	9	1289 -52	156LYS(HZ1)-21THR(OG1)	48.5
	10	31- 1301	20TYR(H) - 157CYS(O)	81.7
	11	58 - 1280	22CYS(H) - 155LEU(O)	84.2
	12	1295 - 47	157CYS(H) - 20 TYR(O)	92.6
Inhibitor-TP	1	53 - 1280	21THR(HG1) - 155LEU(O)	11.7
	2	1289 -29	156LYS(HZ1) - 19 GLY(O)	12.5
	3	1289 -52	156LYS(HZ1)- 21 THR(OG1)	41.3
	4	58 -1280	22CYS(H) - 155LEU(O)	73.5
	5	31 -1301	20TYR(H) - 157CYS(O)	91.1
	6	1295 -47	157CYS(H) - 20 TYR(O)	96.6

Table S4. H-bonds pairs formed between β5 and β6

	No.	H-bonds Pairs	Donor-Acceptor	Occupation (%)
	1	1319 - 1089	159LYS(HZ1) - 135GLN(NE2)	0
	2	1074 - 1345	134THR(H) - 162ILE(O)	0
	3	1319 - 1093	159LYS(HZ1) - 135GLN(O)	0.2
	4	1319 - 1088	159LYS(HZ1) - 135GLN(OE1)	2.2
	5	1090 - 1323	135GLN(E21) - 159LYS(O)	2.3
APO-TP	6	1282 - 1132	156LYS(H) - 140GLY(O)	3.9
	7	1129 - 1293	140GLY(H) - 156LYS(O)	9.1
	8	1125 - 1293	139SER(HG) - 156LYS(O)	27.8
	9	1338 - 1081	162ILE(H) - 134THR(O)	32.1
	10	1095 - 1329	136CYS(H) - 160ALA(O)	77.1
	11	1325 - 1101	160ALA(H) - 136CYS(O)	93.6
	12	1112 - 1310	138ILE(H) - 158LEU(O)	94.2
	13	1303 - 1119	158LEU(H) - 138ILE(O)	96.6
	1	1325 - 1088	160ALA(H) - 135GLN(OE1)	15.1
	2	1090 - 1329	135GLN(E21) - 160ALA(O)	18.3
	3	1319 - 1093	159LYS(HZ1) - 135GLN(O)	18.5
	4	1319 - 1089	159LYS(HZ1) - 135GLN(NE2)	18.5
	5	1125 - 1293	139SER(HG) - 156LYS(O)	19.4
	6	1090 - 1323	135GLN(E21) - 159LYS(O)	19.4
Cu(II)-TP	7	1319 - 1088	159LYS(HZ1) - 135GLN(OE1)	20.1
	8	1282 - 1132	156LYS(H) - 140GLY(O)	23.2
	9	1129 - 1293	140GLY(H) - 156LYS(O)	25.7
	10	1303 - 1119	158LEU(H) - 138ILE(O)	73.3
	11	1338 - 1081	162ILE(H) - 134THR(O)	81.3
	12	1095 - 1329	136CYS(H) - 160ALA(O)	83.3
	13	1325 - 1101	160ALA(H) - 136CYS(O)	88.9
	14	1112 - 1310	138ILE(H) - 158LEU(O)	94.4
	1	1325 - 1088	160ALA(H) - 135GLN(OE1)	0
	2	1319 - 1089	159LYS(HZ1) - 135GLN(NE2)	0
	3	1319 - 1093	159LYS(HZ1) - 135GLN(O)	0.1
	4	1125 - 1293	139SER(HG) - 156LYS(O)	2.9
	5	1319 - 1088	159LYS(HZ1) - 135GLN(OE1)	4
	6	1090 - 1323	135GLN(E21) - 159LYS(O)	4.8
Inhibitor-TP	7	1282 - 1132	156LYS(H) - 140GLY(O)	39.1
	8	1129 - 1293	140GLY(H) - 156LYS(O)	75.6
	9	1338 - 1081	162ILE(H) - 134THR(O)	77.7
	10	1095 - 1329	136CYS(H) - 160ALA(O)	83.4
	11	1325 - 1101	160ALA(H) - 136CYS(O)	91.9
	12	1112 - 1310	138ILE(H) - 158LEU(O)	92
	13	1303 - 1119	158LEU(H) - 138ILE(O)	97.5
